# Use of broad-spectrum antimicrobials for more than 72 h and the detection of multidrug-resistant bacteria in Japanese intensive care units: a multicenter retrospective cohort study

**DOI:** 10.1186/s13756-022-01146-3

**Published:** 2022-09-29

**Authors:** Hideki Yoshida, Takako Motohashi, Liesbet De Bus, Jan De Waele, Akihiro Takaba, Akira Kuriyama, Atsuko Kobayashi, Chie Tanaka, Hideki Hashi, Hideki Hashimoto, Hiroshi Nashiki, Mami Shibata, Masafumi Kanamoto, Masashi Inoue, Satoru Hashimoto, Shinshu Katayama, Shinsuke Fujiwara, Shinya Kameda, Shunsuke Shindo, Taketo Suzuki, Tetsuya Komuro, Toshiomi Kawagishi, Yasumasa Kawano, Yoshihito Fujita, Yoshiko Kida, Yuya Hara, Shigeki Fujitani, DIANA Study Japanese group

**Affiliations:** 1 Department of Emergency and Critical Care Medicine, St. Marianna University School of Medicine, 2-16-1 Sugao, Miyamae, Kawasaki, Kanagawa 216-8511 Japan; 2 Department of Preventive Medicine, St. Marianna University School of Medicine, 2-16-1 Sugao, Miyamae, Kawasaki, Kanagawa Japan; 3 Department of Critical Care Medicine, Ghent University Hospital, C. Heymanslaan 10, 9000 Ghent, Belgium; 4 JA Hiroshima General Hospital, 1-3-3 Jigozen, Hatsukaichi, Hiroshima, Japan; 5 Emergency and Critical Care Center, Kurashiki Central Hospital, 1-1-1 Miwa, Kurashiki, Okayama 710-8602 Japan; 6 Takarazuka City Hospital, 4-5-1 Kohama, Takarazuka, Hyogo Japan; 7 Nippon Medical School Tama Nagayama Hospital, 1-7-1 Nagayama, Tama, Tokyo, Japan; 8 Tokyo Bay Urayasu Ichikawa Medical Center, 3-4-32 Todaijima, Urayasu, Chiba Japan; 9 Hitachi General Hospital, 2-1-1 Jonancho, Hitachi, Ibaraki Japan; 10 Iwate Prefectural Central Hospital, 1-4-1 Ueda, Morioka, Iwate Japan; 11 Department of Emergency and Critical Care Medicine, Wakayama Medical University Hospital, 811-1 Kimiidera, Wakayama, Japan; 12 Department of Anesthesiology and Intensive Care Medicine, Gunma University Hospital, 3-39-15 Showamachi, Maebashi, Gunma Japan; 13 Department of Anesthesiology, Nagoya City University Hospital, 1 Kawasumi, Mizuhochō, Mizuho Ward, Nagoya, Aichi Japan; 14 University Hospital, Kyoto Prefectural University of Medicine, 465 Kajiichō, Kamigyo Ward, Kyoto, Japan; 15 Division of Intensive Care, Department of Anesthesiology and Intensive Care Medicine, Jichi Medical University School of Medicine, 3311-1 Yakushiji, Shimotsuke, Tochigi 329-0498 Japan; 16 National Hospital Organization Ureshino Medical Center, Shimojuku-kou, Ureshino-machi, Ureshino-shi, Saga, 4279-3 Japan; 17 Jikei University School of Medicine Hospital, 3-25-8, Nishi-Shimbashi, Minato-ku, Tokyo, Japan; 18 Omori Red Cross Hospital, 4-30-1 Chuo, Ota Eard, Tokyo, Japan; 19 Yokohama City Minato Red Cross Hospital, 3-12-1 Shinyamashita, Naka Ward, Yokohama, Kanagawa Japan; 20 Shonan Kamakura General Hospital, 1370-1 Okamoto, Kamakura, Kanagawa Japan; 21 Toyama University Hospital, 2630 Sugitani, Toyama-shi, Toyama, Japan; 22 Department of Emergency and Critical Care Medicine, Fukuoka University Hospital, 7-45-1 Nanakuma, Jonan Ward, Fukuoka, Japan; 23 Aichi Medical University Hospital, 1-1 Karimata, Yazako, Nagakute, Aichi Japan; 24 Hiroshima University Hospital, 1-2-3 Kasumi, Minami Ward, Hiroshima, Japan; 25 Yodogawa Christian Hospital, 1-7-50 Kunijima, Higashiyodogawa Ward, Osaka, Japan

**Keywords:** De-escalation, Intensive care units, Multidrug resistance, Broad-spectrum antimicrobials

## Abstract

**Background:**

Large multicenter studies reporting on the association between the duration of broad-spectrum antimicrobial administration and the detection of multidrug-resistant (MDR) bacteria in the intensive care unit (ICU) are scarce. We evaluated the impact of broad-spectrum antimicrobial therapy for more than 72 h on the detection of MDR bacteria using the data from Japanese patients enrolled in the DIANA study.

**Methods:**

We analyzed the data of ICU patients in the DIANA study (a multicenter international observational cohort study from Japan). Patients who received empirical antimicrobials were divided into a broad-spectrum antimicrobial group and a narrow-spectrum antimicrobial group, based on whether they received broad-spectrum antimicrobials for more or less than 72 h, respectively. Differences in patient characteristics, background of infectious diseases and empirical antimicrobial administration, and outcomes between the two groups were compared using the chi-square tests (Monte Carlo method) for categorical variables and the Mann–Whitney U-test for continuous variables. We also conducted a logistic regression analysis to investigate the factors associated with the detection of new MDR bacteria.

**Results:**

A total of 254 patients from 31 Japanese ICUs were included in the analysis, of whom 159 (62.6%) were included in the broad-spectrum antimicrobial group and 95 (37.4%) were included in the narrow-spectrum antimicrobial group. The detection of new MDR bacteria was significantly higher in the broad-spectrum antimicrobial group (11.9% vs. 4.2%, *p* = 0.042). Logistic regression showed that broad-spectrum antimicrobial continuation for more than 72 h (OR [odds ratio] 3.09, *p* = 0.047) and cerebrovascular comorbidity on ICU admission (OR 2.91, *p* = 0.041) were associated with the detection of new MDR bacteria.

**Conclusions:**

Among Japanese ICU patients treated with empirical antimicrobials, broad-spectrum antimicrobial usage for more than 72 h was associated with the increased detection of new MDR bacteria. Antimicrobial stewardship programs in ICUs should discourage the prolonged use of empirical broad-spectrum antimicrobial therapy.

*Trial registration* ClinicalTrials.gov, NCT02920463, Registered 30 September 2016, https://clinicaltrials.gov/ct2/show/NCT02920463

**Supplementary Information:**

The online version contains supplementary material available at 10.1186/s13756-022-01146-3.

## Background

The emergence of drug-resistant bacteria is an urgent issue worldwide. As many as 28 million cases of drug-resistant bacterial infection and 35,000 deaths are reported per year in the United States, and large increases in the prevalence rate of community fecal extended-spectrum β-lactamase (ESBL) carriage have also been reported in other regions [1, 2]. In Japan, an antimicrobial resistance action plan was adopted in 2015. However, the isolation rate of resistant bacteria has not decreased, and the 2020 targets were not reached for methicillin-resistant *Staphylococcus aureus* (MRSA) (47.7% in 2019 vs. the target value of 20% or lower); fluoroquinolone-resistant *Escherichia coli* (41.4% in 2019 vs. the target of 25% or lower); or carbapenem-resistant *Pseudomonas aeruginosa* (10.6% in 2019 vs. the target value of 10% or lower) [3].

In intensive care units (ICUs), broad-spectrum antimicrobials are widely used and there is a high incidence of drug-resistant bacterial infections in critically ill patients. The Extended Study on Prevalence of Infection in Intensive Care III, an international multicenter observational study, found that 54% of ICU patients had infectious diseases and that 70% of patients received antimicrobials [4]. In addition, the EUROBACT 1 study, another international multicenter observational study, found that in 48% of ICU patients, bacteremia was caused by multidrug-resistant (MDR) bacteria [5].

Various attempts have been made to reduce antimicrobial usage in ICUs, including using procalcitonin to initiate or discontinue use of antimicrobials [6, 7], interventions by antimicrobial stewardship teams [8, 9], and antimicrobial discontinuation strategies using scoring systems [10]. Among these strategies, antimicrobial de-escalation plays a central role in antimicrobial stewardship programs (ASP). However, several issues have been identified regarding the de-escalation of antimicrobials. First, there is no worldwide consensus on a definition of “de-escalation,” and different definitions have been used in different studies [11, 12]. Second, no high-quality studies have shown that de-escalation of antimicrobials can reduce the incidence of MDR bacterial infections or improve patient prognosis. One small non-blinded randomized controlled trial found that de-escalation programs had the potential to worsen patient prognosis [13], even though the incidence of *Clostridioides difficile* infection might be reduced by early de-escalation of antipseudomonal β-lactams [14].

The Determinants of Antimicrobial Use and De-escalation in Critical Care (DIANA) study was an international multicenter observational cohort study approved by the European Society of Intensive Care Medicine [15] that followed-up on ICU patients treated with empirical antimicrobials and enrolled 1,495 patients from 152 ICUs in 28 countries worldwide. Only 16.1% of the patients had antimicrobials de-escalated within the first 72 h. The clinical cure rate of patients who had antimicrobials de-escalated within 72 h was higher than that of participants who continued receiving the same antimicrobials for more than 72 h, but there was no difference in the detection of MDR bacteria [15].

In the DIANA study, the definition of de-escalation was “to change the antimicrobial with the intention of the treating physician to narrow the antimicrobial spectrum.” Therefore, de-escalation and discontinuation of broad-spectrum antimicrobials are not synonymous. Furthermore, few studies to date have evaluated the association between the continuation of broad-spectrum antimicrobials and the detection of MDR bacteria in ICUs [12]. Therefore, by using an objective and direct definition of de-escalation, defined by the length of empirical broad-spectrum antimicrobial administration, we evaluated whether broad-spectrum antimicrobial continuation for more than 72 h after the initiation of empirical antimicrobials was associated with the detection of MDR bacteria in the participants from the DIANA study in Japan.

## Methods

### Participants

This multicenter, retrospective cohort study analyzed the data from Japanese participants in the DIANA study [15]. In the DIANA study, patients were recruited from October 2016 until May 2018. Patients were eligible for inclusion in the study if they were aged 18 years or older and admitted to an ICU with an anticipated need of at least 48 h of ICU support. An empirical antimicrobial therapy had to be initiated in the ICU or no more than 24 h prior to ICU admission to treat a community-, healthcare-, hospital- or ICU-acquired bacterial infection. The research protocol was approved by the ethics committee of the Institutional Review Board of St Marianna University School of Medicine (No 5015). The requirement for participant consent was waived because the analysis was retrospective.

Participants with inadequate data on the date of initiation or discontinuation of antimicrobials or inadequate data on the date of MDR bacteria detection after the initiation of empirical antimicrobials, and participants who died within 72 h after enrollment were excluded from the analysis.

**Fig. 1:**
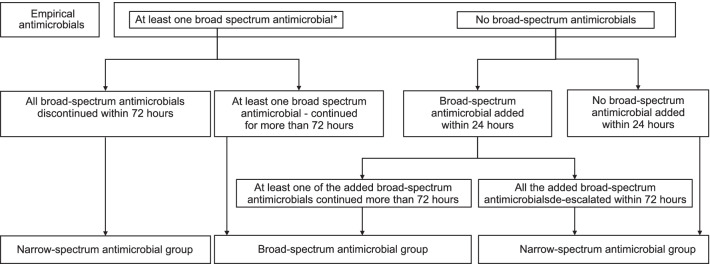
Flowchart of participant assignment to empirical antimicrobial treatment groups according to the empirical antimicrobials administered and the duration of their use. *The definitions of broad-spectrum antimicrobials and narrow-spectrum antimicrobials are provided in Table 1

Patients were divided into a broad-spectrum antimicrobial group and a narrow-spectrum antimicrobial group. The broad-spectrum antimicrobial group included participants who had received at least one broad-spectrum antimicrobial, continued for at least 72 h after the initiation of empirical antimicrobials. Patients initiated on narrow-spectrum empirical antimicrobials in whom at least one broad-spectrum antimicrobial was subsequently added within 24 h and continued for at least 72 h were included in the broad-spectrum antimicrobial group. The remaining participants were assigned to the narrow-spectrum antimicrobial group (Fig. 1).

### Measurements

Data on patient characteristics, background of infectious diseases, and empirical antimicrobials were collected for each patient. Patient characteristic variables included sex, age, disease categories (medical, surgical, and trauma) on ICU admission, ICU admission diagnosis (respiratory, digestive, cardiovascular, neurological, renal and genitourinary, trauma and skin, and other), comorbidities (cardiovascular, diabetes mellitus, solid tumor, renal failure, cerebrovascular, pulmonary, and other), and severity of the condition on ICU admission (Acute Physiology and Chronic Health Evaluation [APACHE] II, Simplified Acute Physiology Score [SAPS] II, and Sequential Organ Failure Assessment [SOFA] scores). Background variables of infectious diseases included health care exposure, immunosuppression, MDR detection by day 2 of the empirical treatment initiation, infectious focus (respiratory tract, gastrointestinal tract, skin and soft tissue, genitourinary tract, catheter-related, other focus, and unknown), septic shock, causative microbial identification, multiple bacterial identifications, bacteremia, need for source control, effective source control by day 3, the timing from the hospital admission to empirical antimicrobial initiation, and the timing from the ICU admission to empirical antimicrobial initiation. Empirical antimicrobial variables included the number of empirical antimicrobials administered, the category of the administered empirical antimicrobials, the duration of empirical antimicrobial administration, and inappropriate administration of empirical antimicrobials.

The primary outcome was new detection of MDR bacteria. The decision on whether and when to take cultures after initiation of the empiric antimicrobials was at the discretion of the physician. Secondary outcomes included clinical cure on day 7, recurrence of infection (by day 28), ICU and in-hospital mortality (by day 28), number of ICU-free days (by day 28), duration of organ support in the ICU (vasopressor use, ventilator use, and renal replacement therapy), number of hospital-free days (by day 28), and number of antimicrobial-free days (by day 28).

### Definitions

The following definitions were used:

#### MDR bacteria

Bacteria that produce ESBL or carbapenemase, *Stenotrophomonas maltophilia*, methicillin-resistant *Staphylococcus aureus* (MRSA), vancomycin-resistant *Enterococcus spp*., or a pathogen resistant to 3 or more antimicrobial classes.

#### Broad-spectrum antimicrobials

Antimicrobials classified as “watch” or “reserve” in the World Health Organization Essential Medicines List Antibiotic Groups [16], and with activity against *Pseudomonas* spp. or anti-MRSA activity (Table 1). Aminoglycosides were also included in the broad-spectrum group. Other antimicrobials were defined as narrow-spectrum antimicrobials.

**Table 1:** Broad-spectrum and narrow-spectrum grouping of antimicrobials

Groups	Category or name
Narrow-spectrum antimicrobials	First-generation cephalosporin
	Second-generation cephalosporin
	Third-generation cephalosporin
	Cefmetazole
	Penicillin
	Penicillin + β-lactamase inhibitor
	Macrolides
	Tetracyclines
	Folate pathway inhibitor
	Nitroimidazoles
	Lincosamide
	Ansamycin
Broad-spectrum antimicrobials	Fourth-generation cephalosporin
	Antipseudomonal penicillin
	Antipseudomonal penicillin + β-lactamase inhibitor
	Carbapenem
	Fluoroquinolone
	Aminoglycoside
	Glycopeptide
	Lipopeptides
	Oxazolidinones

#### New MDR bacteria

MDR bacteria detected on day 2 or later during the 28-day follow-up period and not present before day 2. When no culture was performed after the initiation of empirical antimicrobials, it was assumed that no MDR bacteria were present.

#### Clinical cure

Resolution of all symptoms related to the infection.

#### Recurrence of infection

An infection with the same causative microorganism and source that occurred after discontinuation of all antimicrobial agents for the primary infection.

#### Healthcare exposure

Prior hospitalization within 6 months prior to enrollment, antimicrobial use within 3 months prior to enrollment, nursing home admission, hemodialysis, and invasive procedures within 30 days prior to enrollment, either at home or in an outpatient setting.

#### Immunosuppression

The presence of congenital immunodeficiency, neutropenia (neutrophil count < 1000 cells/µL), use of steroids (> 0.5 mg/kg/day in prednisolone equivalent for > 3 months), solid organ transplantation with immunosuppressant use, bone marrow transplantation with immunosuppressant use, chemotherapy in the year prior to enrollment, radiotherapy in the year prior to enrollment, autoimmune diseases with immunosuppressant use, or human immunodeficiency virus (HIV) infection.

### Statistical analysis

The broad-spectrum and narrow-spectrum antimicrobial groups were compared and the significance of differences between groups was assessed using chi-square tests (Monte Carlo method) or Fisher’s exact tests for categorical variables, and Mann–Whitney U-tests for continuous variables. Two-tailed *p*-values < 0.05 were considered statistically significant.

A univariate analysis was performed using logistic regression to determine the influence of patient characteristics, background of infectious diseases, and empirical antimicrobial usage on the detection of new MDR bacteria and the results were reported as odds ratios (ORs) and 95% confidence intervals (CIs). All analyses were conducted using IBM SPSS Statistics version 24 (IBM, Armonk, NY, USA).

## Results

**Fig. 2:**
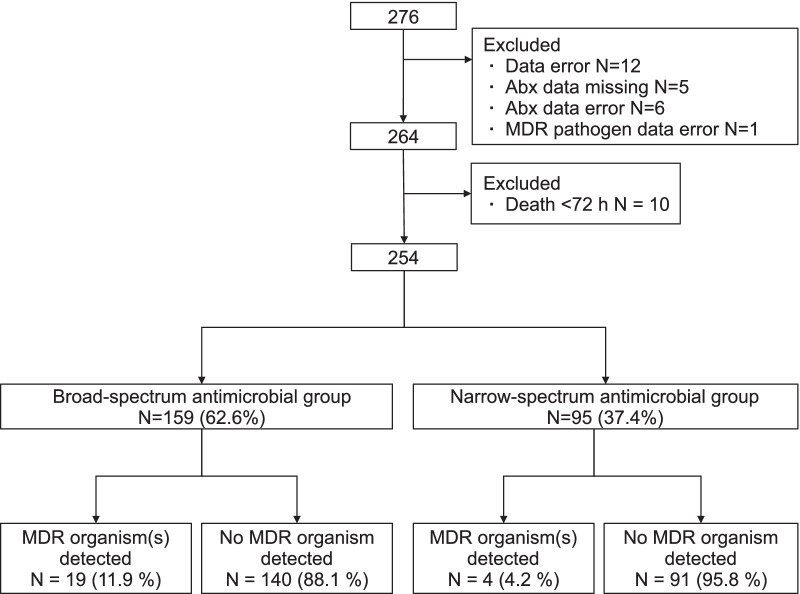
Flowchart of participant selection and retrospective assignment to empirical antimicrobial treatment groups according to the empirical antimicrobials administered and the duration of their use. Abx: antimicrobials, MDR: multidrug-resistant

Of the 276 patients at 31 facilities enrolled in the DIANA study in Japan, 254 (92.0%) patients who met the inclusion criteria were included in the analysis. There were 159 patients (62.6%) in the broad-spectrum antimicrobial group and 95 patients (37.4%) in the narrow-spectrum antimicrobial group (Fig. 2).

The patients in the broad-spectrum antimicrobial group had more severe illness on ICU admission than those in the narrow-spectrum antimicrobial group based on their median APACHE II (21 vs. 17, *p* = 0.002), SAPS (48 vs. 37, *p* < 0.001), and SOFA (8 vs. 5, *p* < 0.001) scores (Table 2).

**Table 2:** Patient characteristics on intensive care unit admission according to the empirical antimicrobial treatment strategy used

	Total	Broad-spectrum antimicrobial group	Narrow-spectrum antimicrobial group	*p* value
	n = 254	n = 159	n = 95	
Age	72 (59–80.25)	73 (62–80)	72 (53–82)	0.401
Sex				0.698
Male	143 (56.3%)	88 (55.3%)	55 (57.9%)	
Female	111 (43.7%)	71 (44.7%)	40 (42.1%)	
Severity on ICU admission				
APACHE II on ICU admission	20 (15–26)	21 (17–27)	17 (13–26)	0.002
SAPS II on ICU admission	43 (29–57)	48 (34–61)	37 (22–50)	< 0.001
Total SOFA score on ICU admission	7 (5–10)	8 (5–11)	5 (4–9)	< 0.001
Admission category				
Medical	175 (68.9%)	109 (68.6%)	66 (69.5%)	> 0.999
Surgical	74 (29.1%)	47 (29.6%)	27 (28.4%)	0.887
Trauma	5 (2.0%)	3 (1.9%)	2 (2.1%)	> 0.999
Admission diagnosis^a^				
Respiratory	80 (31.5%)	46 (28.9%)	34 (35.8%)	0.267
Digestive	68 (26.8%)	53 (33.3%)	15 (15.8%)	0.002
Cardiovascular	62 (24.4%)	36 (22.6%)	26 (27.4%)	0.451
Neurological	36 (14.2%)	17 (10.7%)	19 (20.0%)	0.043
Renal genitourinary	30 (11.8%)	17 (10.7%)	13 (13.7%)	0.548
Trauma skin	21 (8.3%)	12 (7.5%)	9 (9.5%)	0.641
Other	26 (10.2%)	16 (10.1%)	10 (10.5%)	> 0.999
Comorbidities (N = 241)				
Cardiovascular	58 (24.1%)	38 (25.0%)	20 (22.5%)	0.755
Diabetes mellitus	54 (22.4%)	35 (23.0%)	19 (21.3%)	0.873
Solid tumor	40 (16.6%)	28 (18.4%)	12 (13.5%)	0.372
Renal failure	31 (12.9%)	22 (14.5%)	9 (10.1%)	0.426
Cerebrovascular	31 (12.9%)	24 (15.8%)	7 (7.9%)	0.109
Pulmonary	26 (10.8%)	18 (11.8%)	8 (9.0%)	0.528
Other	18 (7.5%)	15 (9.9%)	3 (3.4%)	0.077

Results are shown as n (%) or median (IQR) where applicable

*ICU* intensive care unit, *APACHE* Acute Physiology and Chronic Health Evaluation, *SAPS* Simplified Acute Physiology Score, *SOFA* Sequential Organ Failure Assessment

^a^Patients could have multiple admission diagnoses

Compared with the narrow-spectrum antimicrobial group, the broad-spectrum antimicrobial group had a significantly higher incidence of healthcare exposure (49.0% vs. 33.7%, *p* = 0.023), incidence of septic shock (32.1% vs. 17. 9%, *p* = 0.019), SOFA score on day 0 (median: 8 vs. 6, *p* = 0.001), and longer time from the hospital admission to the empirical antimicrobial initiation (Table 3).

**Table 3:** Infection-related characteristics according to the empirical antimicrobial treatment strategy used

	Total	Broad-spectrum antimicrobial group	Narrow-spectrum antimicrobial group	*p* value
Health care exposure (N = 244)	106 (43.4%)	76 (49.0%)	30 (33.7%)	0.023
Hospitalization for ≥ 2 days in the 12 months prior to study inclusion	56 (23.0%)	38 (24.5%)	18 (20.2%)	0.528
Antimicrobial exposure in the 3 months prior to study inclusion	45 (18.4%)	35 (22.6%)	10 (11.2%)	0.039
Resident in a nursing home or long-term care facility	19 (7.8%)	13 (8.4%)	6 (6.7%)	0.805
Receiving invasive procedures at home^a^	17 (7.0%)	9 (5.8%)	8 (9.0%)	0.434
Chronic hemodialysis^a^	11 (4.5%)	8 (5.2%)	3 (3.4%)	0.750
Immunosuppressed status (N = 248)^b^	34 (13.7%)	25 (15.9%)	9 (9.9%)	0.250
Baseline MDR colonization (N = 253)^c^	22 (8.7%)	18 (11.3%)	4 (4.3%)	0.065
Source of infection^d^				
Respiratory tract	89 (35.0%)	49 (30.8%)	40 (42.1%)	0.078
Gastrointestinal tract	59 (23.2%)	45 (28.3%)	14 (14.7%)	0.014
Skin soft tissue	24 (9.4%)	14 (8.8%)	10 (10.5%)	0.663
Genitourinary tract	24 (9.4%)	12 (7.5%)	12 (12.6%)	0.190
Catheter-related	9 (3.5%)	6 (3.8%)	3 (3.2%)	> 0.999
Other focus	13 (5.1%)	9 (5.7%)	4 (4.2%)	0.772
Unknown	41 (16.1%)	24 (15.1%)	17 (17.9%)	0.599
Septic shock	68 (26.8%)	51 (32.1%)	17 (17.9%)	0.019
SOFA day 0	7 (4–10)	8 (5–11)	6 (3–9)	0.001
SOFA day 3	5 (3–8)	6 (3–9)	4 (2–6)	< 0.001
Microbiologically documented infection	126 (49.6%)	82 (51.6%)	44 (46.3%)	0.439
Polymicrobial infection	35 (13.8%)	24 (15.1%)	11 (11.6%)	0.459
Bacteremia	61 (24.0%)	43 (27.0%)	18 (18.9%)	0.172
Need for source control	75 (29.5%)	54 (34.0%)	21 (22.1%)	0.048
Effectiveness of source control on day 3^e^	63 (84.0%)	43 (79.6%)	20 (95.2%)	0.952
From hospital admission to empirical antimicrobial initiation	1 (1–4)	1 (1–10)	1 (1–1)	< 0.001
From ICU admission to empirical antimicrobial initiation	1 (0–1)	1 (1–1)	1 (1–1)	0.202

Results are shown as n (%) or median (IQR) where applicable

*SOFA* Sequential Organ Failure Assessment

^a^In the last 30 days prior to study inclusion

^b^The presence of congenital immunodeficiency, neutropenia (absolute neutrophil count < 1000 cells/μL), patient receiving corticosteroid treatment (prednisolone or equivalent > 0.5 mg/kg/day for > 3 months prior to study inclusion), solid organ transplant patient receiving immunosuppressive treatment, bone marrow transplant patient receiving immunosuppressive treatment, administration of chemotherapy in the year prior to enrollment, radiotherapy in the year prior to enrollment, autoimmune disease with the use of an immunosuppressive treatment, or human immunodeficiency virus (HIV) infection in the subgroup of patients with data available

^c^Defined as all MDR pathogens presumed to be already present on ICU admission, within 1 year prior to study inclusion combined with all MDR pathogens not present on ICU admission and detected before day 2 (day 0 is considered start date of the empiric antimicrobial therapy) in the subgroup of patients with data available

^d^Patients could have multiple infection diagnoses

^e^n = number of patients who need source control

Compared with the narrow-spectrum antimicrobial group, initiation of the following broad-spectrum antimicrobials was more frequent in the broad-spectrum antimicrobial group: carbapenems (50.9% vs. 12.6%, *p* < 0.001), antipseudomonal penicillin + β-lactamase inhibitors (36.5% vs. 11.6%, *p* < 0.001), and glycopeptides (23.9% vs. 8%, *p* = 0.002) (Table 4). The duration of antimicrobial therapy was also significantly longer in the broad-spectrum antimicrobial group compared to the narrow-spectrum antimicrobial group (median: 12 days vs. 7 days, *p* < 0.001).

**Table 4:** Antimicrobial characteristics of study-related infections according to the empirical antimicrobial treatment strategy used

	Total	Broad-spectrum antimicrobial group	Narrow-spectrum antimicrobial group	*p* value
Mono/polytherapy				
Monotherapy	178 (70.1%)	104 (65.4%)	74 (77.9%)	0.047
Combination therapy	76 (29.9%)	55 (34.6%)	21 (22.1%)	0.047
2 Antimicrobial agents	58 (22.8%)	43 (27.0%)	15 (15.8%)	0.045
≥ 3 Antimicrobial agents	18 (7.1%)	12 (7.5%)	6 (6.3%)	0.805
Antimicrobial types				
Carbapenem	93 (36.6%)	81 (50.9%)	12 (12.6%)	< 0.001
Antipseudomonal penicillin + β-lactamase inhibitor	69 (27.2%)	58 (36.5%)	11 (11.6%)	< 0.001
Glycopeptide	46 (18.1%)	38 (23.9%)	8 (8.4%)	0.002
Penicillin + β-lactamase inhibitor	40 (15.7%)	4 (2.5%)	36 (37.9%)	< 0.001
Third-generation cephalosporin	28 (11.0%)	9 (5.7%)	19 (20.0%)	0.001
First-generation cephalosporin	14 (5.5%)	1 (0.6%)	13 (13.7%)	< 0.001
Macrolide	12 (4.7%)	6 (3.8%)	6 (6.3%)	0.373
Fluoroquinolone	10 (3.9%)	8 (5.0%)	2 (2.1%)	0.329
Second-generation cephalosporin	7 (2.8%)	0 (0.0%)	7 (7.4%)	0.001
Penicillin	5 (2.0%)	4 (2.5%)	1 (1.1%)	0.653
Lincosamide	5 (2.0%)	2 (1.3%)	3 (3.2%)	0.366
Aminoglycoside	4 (1.6%)	4 (2.5%)	0 (0.0%)	0.300
Oxazolidinone	4 (1.6%)	1 (0.6%)	3 (3.2%)	0.149
Fourth-generation cephalosporin	3 (1.2%)	3 (1.9%)	0 (0.0%)	0.295
Lipopeptide	3 (1.2%)	3 (1.9%)	0 (0.0%)	0.295
Others	7 (2.8%)	6 (3.8%)	1 (1.1%)	0.262
Duration of treatment for the infection under study (days)	10.5 (6–16)	12 (7–18)	7 (5–13)	< 0.001
Inappropriate empirical antimicrobial prescription^a^	12 (4.7%)	8 (5.0%)	4 (4.2%)	> 0.999

Results are shown as n (%)

^a^Presence of a causative pathogen resistant to the initial agent(s) leading to addition or replacement of the empirical antimicrobial agent

The detection of new MDR bacteria was significantly higher in the broad-spectrum antimicrobial group than in the narrow-spectrum antimicrobial group (11.9% vs. 4.2%, *p* = 0.042) (Table 5). Compared with the narrow-spectrum antimicrobial group, patients in the broad-spectrum antimicrobial group had significantly fewer hospital-free days by day 28 (median, (interquartile range [IQR]): (0, [0–3] days vs. 0, [0–15] days, *p* = 0.011) and significantly fewer antimicrobial-free days by day 28 (median 13 days vs. 17 days, *p* = 0.001) among the patients alive at day 28. The clinical cure at day 7, the recurrence of infection, the ICU mortality by day 28, and the in-hospital mortality by day 28 were not significantly different between the two groups.

**Table 5:** Patient outcomes according to the empirical antimicrobial treatment strategy used

	Total	Broad-spectrum antimicrobial group	Narrow-spectrum antimicrobial group	*p* value
Emergence of new MDR pathogens^a^	23 (9.1%)	19 (11.9%)	4 (4.2%)	0.042
Clinical cure on day 7	135 (53.1%)	88 (55.3%)	47 (49.5%)	0.436
ICU mortality^a^	18 (7.1%)	14 (8.8%)	4 (4.2%)	0.211
28-day mortality (n = 253)	31 (12.3%)	23 (14.6%)	8 (8.4%)	0.170
Number of days in the ICU (N = 236)^a, b^	7 (4–15)	7 (4–15)	7 (3–16)	0.707
On vasoactive drugs	2 (0–5)	3 (1–6)	1 (0–5)	0.001
On Invasive mechanical ventilation	3 (0–8)	4 (0–9)	2 (0–5)	0.015
Receiving renal replacement therapy	0 (0–0)	0 (0–0)	0 (0–0)	0.026
ICU-free days (N = 221)^a, c^	21 (11–24)	21 (10–24)	21 (11–25)	0.529
Hospital-free days (N = 221)^a, c^	0 (0–9)	0 (0–3)	0 (0–15)	0.013
Antimicrobial-free days (N = 221)^a, c^	14 (5–20)	13 (3–18)	17 (8–21)	0.001
Infection relapse^a^	14 (5.6%)	7 (4.5%)	7 (7.4%)	0.398

Results are shown as n (%) or median (IQR) where applicable

^a^Measured from day 2 or later during the 28-day follow-up period and not present before day 2

^b^In the subgroup of intensive care unit (ICU) survivors

^c^In the subgroup of patients alive at day 28

**Fig. 3:**
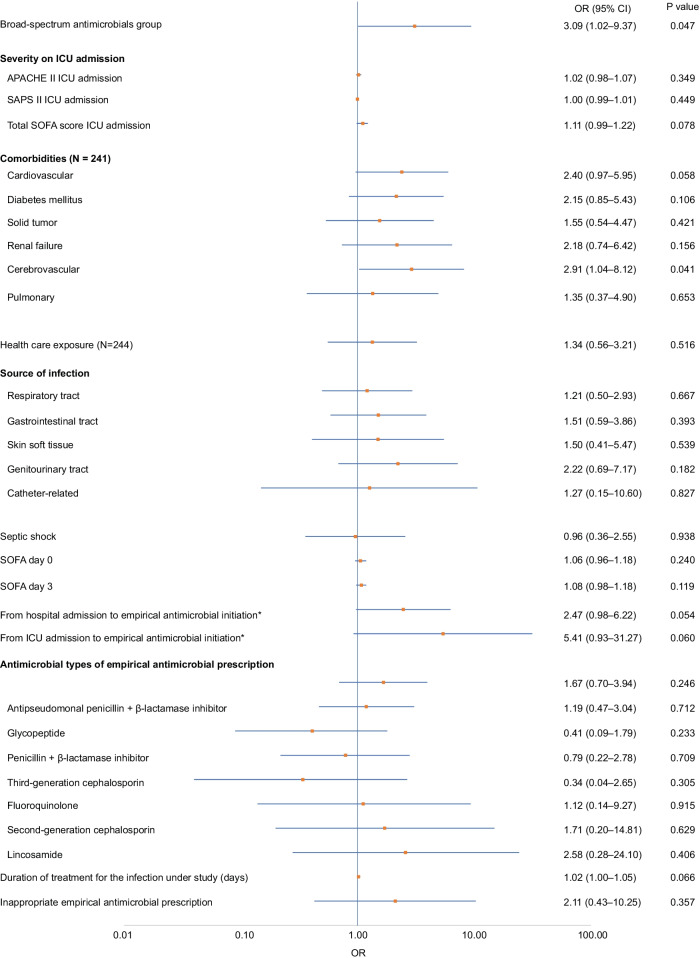
The summary logistic regression analysis for the factors associated with the detection rate of new multidrug-resistant bacteria within 28 days after study enrollment. *The variables of “From hospital admission to empirical antimicrobial initiation” and “From ICU admission to empirical antimicrobial initiation” are the categorical variables grouped into two groups. One group is less than and equal to 7 days from Hospital/ICU admission, the other is more than 7 days. OR: odds ratio; ICU: intensive care unit

Logistic regression analysis showed that broad-spectrum antimicrobial continuation for more than 72 h (OR: 3.09, 95% CI: 1.02–9.37, *p* = 0.047) and cerebrovascular comorbidity on ICU admission (OR: 2.91, 95% CI: 1.04–8.12, *p* = 0.041) were associated with a significantly higher detection rate of new MDR bacteria. The other variables had no significant differences (Fig. 3). Factors associated with the detection rate of new multidrug-resistant bacteria within 28 days after study enrollment are shown in Additional file 1.

## Discussion

The study results revealed that continuation of broad-spectrum antimicrobials for more than 72 h was associated with increased detection of new MDR bacteria. Laurence et al. [17] evaluated the relationship between the administration of imipenem and the detection of imipenem-resistant bacteria in ICU patients and showed that the median time to the development of resistant bacteria was 15 days. However, they found that the risk of developing imipenem-resistant bacteria increased 5.9-fold with the use of imipenem for 72 h or less compared to patients without any imipenem use, and 7.8-fold with the use of imipenem for longer periods compared to patients with imipenem use for less than 72 h [17]. Teshome et al. [18] evaluated the correlation between the duration of exposure to antipseudomonal β-lactam antimicrobials and the development of new resistance in critically ill patients in a large single-center cohort study. They found that each additional day of exposure to any antipseudomonal β-lactam (cefepime, meropenem, or piperacillin-tazobactam) resulted in an adjusted hazard ratio of 1.04 (95% CI: 1.04–1.05) for the development of new antipseudomonal β-lactam resistance. Our study, which included empirical treatment with multiple classes of broad-spectrum antimicrobials in a multicenter study cohort, also showed an increased risk of the detection of MDR bacteria associated with the use of broad-spectrum antimicrobials for more than 72 h. Our results raise the question of whether discontinuing the administration of broad-spectrum antimicrobials within 72 h could reduce the incidence of new MDR bacterial infections. This should be evaluated in future research.

No association was found between the class of antimicrobial agents used as empirical antimicrobial therapy and the detection of new MDR bacteria in our analysis. ICUs manage patients who are critically ill, immunocompromised, or who have deteriorated in other units after receiving antimicrobials, and these patients often require the initiation of empirical broad-spectrum antimicrobial therapy. Multiple guidelines also suggest that early broad-spectrum antimicrobial administration is an important component of sepsis treatment [19, 20]. Therefore, it would be inadvisable to initiate empirical antimicrobial therapy with narrow-spectrum antimicrobials rather than broad-spectrum antimicrobials routinely as part of ASPs. In real-world practice, as our findings showed, a more practical strategy in ICU settings would be to focus on earlier strict discontinuation of broad-spectrum antimicrobials, rather than to prohibit the initiation of broad-spectrum antimicrobials.

The rationale of the continuation of broad-spectrum antimicrobials for more than 72 h was elucidated by comparing the background information of the broad-spectrum and narrow-spectrum antimicrobial groups. The broad-spectrum antimicrobial group had significantly higher illness severity scores on ICU admission and SOFA scores on initiation of empirical antimicrobials. There was also a high proportion of healthcare exposures prior to the initiation of empirical antimicrobial therapy. Septic shock, which reflects severe disease, and use of intravenous antimicrobials within the past 90 days have been reported as risk factors for MDR bacterial infections [21], and broad-spectrum antimicrobials have been recommended as empirical therapies. It is understandable for clinicians to feel that there is risk in discontinuing broad-spectrum antimicrobials, especially if the causative organism cannot be identified for patients with a high risk of MDR infection. Therefore, the results of this study suggest that it would be useful to conduct further research to evaluate the potential benefit of discontinuing broad-spectrum antimicrobials within 72 h in patients at high risk for MDR-resistant infections. Furthermore, it may be efficient to combine such research with an ASP intervention.

Of note, this study used a different definition of de-escalation than that used in the original DIANA study to evaluate the effect of longer broad antimicrobial usage. In the DIANA study, the definition of de-escalation was “to change the antimicrobial with the intention of the treating physician to narrow the antimicrobial spectrum.” However, in this definition, the de-escalation group included patients who changed from broad-spectrum to narrower broad-spectrum antimicrobials and discontinued only one broad-spectrum antimicrobial after initial empirical treatment with more than one broad-spectrum antimicrobial. Additionally, in the original DIANA study, patients in whom empirical antimicrobial therapy was initiated with narrow-spectrum antimicrobials and who did not require de-escalation were included in the non-de-escalation group. There is currently no standard definition of de-escalation, which makes it difficult to evaluate the effectiveness of de-escalation [12]. The essence of de-escalation is to reduce the use of broad-spectrum antimicrobials to reduce the occurrence of MDR organisms. Therefore, a more objective and reproducible definition, defined by whether antipseudomonal antimicrobials and anti-MRSA antimicrobials were stopped within 72 h or not, as in this study, is useful for evaluating the effectiveness of discontinuing broad-spectrum antimicrobial therapy within 72 h from the initiation of empirical antimicrobials.

This study has several limitations. First, the number of cases of infection with new MDR bacteria was relatively small. Therefore, a multivariable analysis could not be conducted to adjust for confounding factors. This meant that we could not show a definitive relationship between the use of broad-spectrum antimicrobials for more than 72 h and the detection of MDR bacteria. Nevertheless, to the best of our knowledge, this study is the first to find such a relationship in Japanese ICUs in a univariable analysis.

Second, the screening for new MDR bacteria was not routinely conducted on all patients; thus, some cases of MDR bacterial occurrence may have been missed. However, both the broad-spectrum and the narrow-spectrum antimicrobial groups were comparable in this regard. In addition, it is likely that the clinically important cases of new MDR bacteria were detected in this study, because cultures were usually taken when an exacerbation of infection and/or new infection was suspected. We also acknowledge that colonization is an important problem that needs to be considered. As all patients enrolled in this study received empirical antimicrobials, it was assumed that most patients had cultures performed at the time of study enrollment and that they were not colonized with the new MDR bacteria detected after study enrollment.

Third, the results cannot be generalized outside Japan because all participants were admitted to ICUs in Japan. However, the available antimicrobials, methods of antimicrobial use, and epidemiology of pathogenic microorganisms vary according to region. Even within the same hospital, ICU patients tend to have a higher proportion of drug-resistant infections than patients in other units [22]. The Japanese data were chosen because Japan had the largest number of participants in the DIANA study from any single country. The Japanese data are unique because of the extremely low de-escalation rate in the DIANA study, which is one of the lowest de-escalation rates in a developed country [15]. The de-escalation rates were 16% in all countries, 13.1% in Japan, 62.2% in the United States, 50% in New Zealand, and 41.7% in Australia. The participating centers had a higher mean number of beds per ICU than the average Japanese hospital (12.5 versus 7 [23]); thus, our results reflect the situation in major Japanese acute care hospitals.

Last, we did not evaluate environmental factors, including the structure of the ICU beds (single room or not) and how well the ICU staff adhered to infection prevention and control measures. However, the new MDR bacteria detected as the primary outcome of this study were different species within each facility, with only one exception. In the facility where two cases of MRSA were detected, the isolates were cultured 3 months apart, ruling out the possibility of direct transmission, suggesting that environmental factors did not have an appreciable effect on the study results.

## Conclusions

In conclusion, there was a higher detection rate of new MDR bacteria in Japanese ICU patients in whom empirical broad-spectrum antimicrobials were administered for more than 72 h than in patients who received narrow-spectrum empirical antimicrobial therapy. More well-designed studies are needed to determine if interventions that restrict the administration of broad-spectrum antimicrobials to less than 72 h reduce the detection of new MDR organisms and/or the incidence of infections caused by MDR organisms, when microbiologically and clinically justified, without worsening patient clinical outcomes. These results provide important evidence for developing ASP interventions in critically ill populations.

## Supplementary Information

**Additional file 1**: Factors associated with the detection rate of new multidrug-resistant bacteria within 28 days after study enrollment. *OR* odds ratio. The result of the logistic regression analysis to investigate the factors associated with the detection rate of new multidrug-resistant bacteria within 28 days after study enrollment.

## Data Availability

The datasets used and/or analyzed during the current study are available from the corresponding author on reasonable request. The DIANA study, evaluating the total data including the Japanese data, was published in Intensive Care Med (PMID: 32,519,003, NCT: 02,920,463), and the original data of our study are owned by the DIANA study Principal Investigator, Jan De Waele.

## Abbreviations

APACHE II: Acute Physiology and Chronic Health Evaluation II
ASP: Antimicrobial stewardship program
CI: Confidence interval
DIANA study: Determinants of Antimicrobial Use and De-escalation in Critical Care study
ESBL: Extended-spectrum β-lactamase
HIV: Human immunodeficiency virus
ICU: Intensive care unit
IQR: Interquartile range
MDR: Multidrug-resistant
MRSA: Methicillin-resistant *Staphylococcus aureus*
OR: Odds ratio
SAPS: Simplified Acute Physiology Score
SOFA: Sequential Organ Failure Assessment
