# Supplementary material for: Use of broad-spectrum antimicrobials for more than 72 h and the detection of multidrug-resistant bacteria in Japanese intensive care units: a multicenter retrospective cohort study
Source: Antimicrob Resist Infect Control. 2022 Sep 29;11:119. doi: 10.1186/s13756-022-01146-3 (PMC9520832; doi:10.1186/s13756-022-01146-3)
Supplement: Supplementary file 1 — Additional file 1: Factors associated with the detection rate of new multidrug-resistant bacteria within 28 days after study enrollment. OR odds ratio. The result of the logistic regression analysis to investigate the factors associated with the detection rate of new multidrug-resistant bacteria within 28 days after study enrollment. [file 13756_2022_1146_MOESM1_ESM.docx]

**Additional file 1.** Factors associated with the detection rate of new multidrug-resistant bacteria within 28 days after study enrollment

|  | OR (95% CI) | p value |
| --- | --- | --- |
| Broad-spectrum antibiotic group or narrow-spectrum antibiotic group | 3.09 (1.02–9.37) | 0.047 |
| Age | 1.02 (0.99–1.05) | 0.159 |
| Male sex | 1.20 (0.51–2.83) | 0.676 |
| **Severity on ICU admission** |  |  |
| APACHE II ICU admission | 1.02 (0.98–1.07) | 0.349 |
| SAPS II ICU admission | 1.00 (0.99–1.01) | 0.449 |
| Total SOFA score ICU admission | 1.11 (0.99–1.22) | 0.078 |
| **Admission category** |  |  |
| Medical | 1.04 (0.41–2.63) | 0.942 |
| Surgical | 1.07 (0.42–2.72) | 0.886 |
| **Admission diagnosis** |  |  |
| Respiratory | 0.75 (0.28–1.98) | 0.559 |
| Digestive | 1.52 (0.61–3.77) | 0.366 |
| Cardiovascular | 1.40 (0.55–3.58) | 0.482 |
| Renal genitourinary | 0.69 (0.15–3.11) | 0.629 |
| Trauma skin | 1.78 (0.48–6.55) | 0.389 |
| **Comorbidities (N=241)** |  |  |
| Cardiovascular | 2.40 (0.97–5.95) | 0.058 |
| Diabetes mellitus | 2.15 (0.85–5.43) | 0.106 |
| Solid tumor | 1.55 (0.54–4.47) | 0.421 |
| Renal failure | 2.18 (0.74–6.42) | 0.156 |
| Cerebrovascular | 2.91 (1.04–8.12) | 0.041 |
| Pulmonary | 1.35 (0.37–4.90) | 0.653 |
| Other | 1.27 (0.27–5.92) | 0.762 |
| **Health care exposure (N=244)** | 1.34 (0.56–3.21) | 0.516 |
| Hospitalization for ≥ 2 days in the 12 months prior to study inclusion | 1.29 (0.48–3.47) | 0.614 |
| Antimicrobial exposure in the last 3 months prior to study inclusion | 0.98 (0.32–3.05) | 0.974 |
| Resident in a nursing home or long-term care facility | 0.54 (0.07–4.25) | 0.558 |
| Receiving invasive procedures at home | 1.38 (0.29–6.47) | 0.683 |
| Chronic hemodialysis | 1.01 (0.12–8.28) | 0.993 |
| Immunosuppressed status (N=248) | 0.57 (0.13–2.57) | 0.468 |
| Baseline colonization (N=253) | 2.48 (0.76–8.08) | 0.132 |
| **Source of infection** |  |  |
| Respiratory tract | 1.21 (0.50–2.93) | 0.667 |
| Gastrointestinal tract | 1.51 (0.59–3.86) | 0.393 |
| Skin soft tissue | 1.50 (0.41–5.47) | 0.539 |
| Genitourinary tract | 2.22 (0.69–7.17) | 0.182 |
| Catheter-related | 1.27 (0.15–10.60) | 0.827 |
| Septic shock | 0.96 (0.36–2.55) | 0.938 |
| SOFA day 0 | 1.06 (0.96–1.18) | 0.240 |
| SOFA day 3 | 1.08 (0.98–1.18) | 0.119 |
| Microbiologically documented infection | 2.51 (1.00–6.34) | 0.051 |
| Polymicrobial infection | 0.57 (0.13–2.55) | 0.464 |
| Bacteremia | 1.13 (0.42–3.01) | 0.807 |
| Need for source control | 1.61 (0.66–3.89) | 0.293 |
| Effectiveness of source control on day 3 (n = number of patients who need source control) | 0.63 (0.11–3.45) | 0.590 |
| From hospital admission to empirical antimicrobial initiation  (Less than or equal to 7 days or not) * | 2.47 (0.98­–6.22) | 0.054 |
| From ICU admission to empirical antimicrobial initiation  (Less than or equal to 7 days or not) * | 5.41 (0.93–31.27) | 0.060 |
| **Treatment characteristics** |  |  |
| **Empirical antimicrobial prescription** |  |  |
| Monotherapy | 2.15 (0.71–6.55) | 0.178 |
| 2 Antimicrobial agents | 0.69 (0.23–2.12) | 0.516 |
| **Antimicrobial types** |  |  |
| Carbapenem | 1.67(0.70–3.94) | 0.246 |
| Antipseudomonal penicillin + β-lactamase inhibitor | 1.19 (0.47–3.04) | 0.712 |
| Glycopeptide | 0.41 (0.09–1.79) | 0.233 |
| Penicillin + β-lactamase inhibitor | 0.79 (0.22–2.78) | 0.709 |
| Third-generation cephalosporin | 0.34 (0.04–2.65) | 0.305 |
| Fluoroquinolone | 1.12 (0.14–9.27) | 0.915 |
| Second-generation cephalosporin | 1.71 (0.20–14.81) | 0.629 |
| Lincosamide | 2.58 (0.28–24.10) | 0.406 |
| Duration of treatment for the infection under study (days) | 1.02 (1.00–1.05) | 0.066 |
| Inappropriate empirical antimicrobial prescription | 2.11 (0.43–10.25) | 0.357 |

* The timing from intensive care unit (ICU) admission to the start of empirical antimicrobials and the timing from the hospital admission to the start of empirical antimicrobials were categorized into two categorical variables. One variable is less than and equal to 7 days from ICU/hospital admission, the other is more than 7 days.

APACHE II, Acute Physiologic Assessment and Chronic Health Evaluation II; CI, confidence interval; OR, odds ratio; SAPS, Simplified Acute Physiology Score; SOFA, Sequential Organ Failure Assessment
